# From Static Choices to Navigating Clinical Trajectories: Reframing Therapeutic Reasoning in Medical Education

**DOI:** 10.1111/jep.70709

**Published:** 2026-11-03

**Authors:** Yskert von Kodolitsch, Tilman Dreyer, Thomas Wick, Sigrid Harendza

**Affiliations:** ^1^ Department of Thoracic and Cardiovascular Surgery Heart and Diabetes Center NRW Ruhr University Bochum Bad Oeynhausen Nordrhein Westphalen Germany; ^2^ Clinic for Cardiology, Pulmonology, Angiology, and Intensive Care Medicine Marienkrankenhaus Hamburg Hamburg Germany; ^3^ Institute of Applied Mathematics Leibniz University Hannover Niedersachesen Germany; ^4^ III. Department of Medicine University Medical Center Hamburg‐Eppendorf Hamburg Hamburg Germany

## Abstract

**Problem:**

Therapeutic reasoning is central to clinical practice and informed by established frameworks such as evidence‐based medicine and shared decision‐making. In educational settings, however, therapeutic decision‐making is frequently taught and assessed as a discrete choice among predefined treatment options. This static framing – commonly structured through decision trees and guideline algorithms– emphasizes treatment selection under uncertainty but may insufficiently reflect the temporally evolving and regulatory nature of patient care.

**Conceptual Shift:**

We propose that therapeutic reasoning can be more fully understood as the navigation of clinical trajectories rather than the selection of isolated choices. Drawing on complex adaptive systems theory, we conceptualize disease, resilience, and health as dynamically interacting functions evolving through feedback loops and shifting stability. Therapeutic reasoning is the anticipatory and interpretive process that guides the adaptive regulation of evolving clinical trajectories across coupled systems of organism and pathology. This perspective complements established decision‐making models by making the temporal and systemic dimensions of therapeutic practice explicit.

**Framework:**

We operationalize this reframing through a phase‐based model distinguishing disease‐dominant decline, therapy‐dominant modulation, and resilience‐dominant recovery. Using severe pneumonia as an illustrative case, we demonstrate how inflection points and destabilizing events reveal the dynamic cognitive work underlying therapeutic practice and provide a conceptual scaffold for structuring therapeutic thinking.

**Implications:**

Reframing therapeutic reasoning as trajectory navigation enriches the teaching of therapeutic decision making by providing a conceptual scaffold for curriculum design, clinical supervision, and assessment.

## 1 The Need for a Dynamic Model of Therapeutic Reasoning

Since Hippocrates, medical education has emphasized decision‐making as central to patient care [1]. Three core domains of medical decision‐making are commonly distinguished: ethics, diagnostics, and therapy. Ethical reasoning has evolved toward shared decision‐making models that integrate medical expertise with patients' values and preferences [2]. Diagnostic reasoning has been extensively theorized and empirically studied as the cognitive process underlying disease identification [3, 4]. Therapeutic reasoning is often perceived as having the greatest impact on patients [5, 6, 7]. However, many researchers argue that teaching in this domain faces complex challenges related to therapeutic decision‐making that remain difficult to capture in comprehensive models [5, 6, 8], and research on the cognitive processes of management reasoning is less well represented in the literature [9]. To date, teaching commonly frames therapy as a discrete choice among predefined alternatives – whether to treat, which treatment to select, and how long to continue treatment. Guidelines and standard operating procedures frequently follow a command‐based paradigm [10], and evidence‐based medicine translates population‐level data into condition‐specific recommendations [11, 12, 13].

This static “choice architecture” of therapeutic reasoning risks conveying that therapeutic decisions are isolated events rather than components of an evolving processes [14]. Yet clinical reality often contradicts this framing: patients may deteriorate despite initially appropriate choices, improvement may lag behind treatment, and therapy itself may destabilize the organism. Rather than merely leaving a conceptual gap, this framing narrows the cognitive target of therapeutic reasoning to option selection, thereby obscuring its fundamentally temporal and regulatory nature. We argue that what is missing is a conceptual model that frames therapeutic reasoning not as a static selection of choices, but as the navigation of evolving clinical trajectories.

In line with approaches to conceptual article design that emphasize theory synthesis and model development [15], this paper integrates insights from complex adaptive systems theory with scholarship on clinical reasoning to propose a reframed understanding of therapeutic reasoning.

## 2 The Particular Demands of Therapeutic Reasoning

Therapeutic reasoning has distinct requirements with specific cognitive targets, temporal structures, and epistemic demands [9]. Primarily, therapeutic reasoning is regulatory rather than classificatory [16]. Its goal transcends the mere identification of a medical condition; instead, it seeks to influence the direction, velocity, and stability of an evolving clinical trajectory. Consequently, therapeutic reasoning unfolds longitudinally, remaining operative even after uncertainty about a diagnosis or immediate therapeutic effects have been reduced [16]. Rather than converging toward a fixed endpoint, therapeutic reasoning requires continuous monitoring, recalibration, and reprioritization in response to changing system states. It spans extended time horizons and demands the anticipation of delayed effects, feedback loops, and potential phase transitions [16]. Furthermore, it must account for the complexities of clinical dynamics. Clinical improvement may lag behind intervention, deterioration may occur despite initially appropriate therapeutic decisions, and therapeutic actions themselves may destabilize the system or shift it into an unintended state.

The epistemic profile of therapeutic reasoning demands tolerance of irreducible uncertainty regarding system behavior over time [8]. Because interventions alter the very system they aim to stabilize, clinicians must reason under conditions in which observation and action are interdependent. They must anticipate nonlinear dynamics, recognize early warning signals of instability, and adapt goals as trajectories evolve [17]. The nature of error is also distinctive. In therapeutic reasoning, failure may arise not only from selecting the wrong intervention but also from misjudging trajectory direction, overlooking tipping points, persisting too long with a once‐appropriate strategy, or failing to shift from disease attenuation to resilience support when system conditions change [18]. Therefore, therapeutic reasoning represents a distinct, temporally embedded, regulatory form of clinical reasoning that warrants explicit theoretical articulation and educational attention [19, 20, 21, 22].

## 3 Two Modes of Therapeutic Reasoning

Therapeutic decision‐making has historically been approached from two major traditions that differ not only in their methodological application but also in their underlying epistemological foundations (Figure 1). The first of these traditions roots in classical medical decision analysis [23]. Within this framework, decisions are represented as decision trees composed of decision nodes (points at which choices are made), chance nodes (uncertain outcomes), and terminal nodes (final outcomes such as death, cure, or cost). In this paradigm, dynamic decisions are conceptualized as sequences of predefined choices within the tree, guided by “if–then” rules that shape evolving treatment strategies [24, 25]. Consequently, clinical reality is modeled as a discrete set of branching pathways among a finite array of alternatives (Figure 1, upper panel).

**Figure 1:**
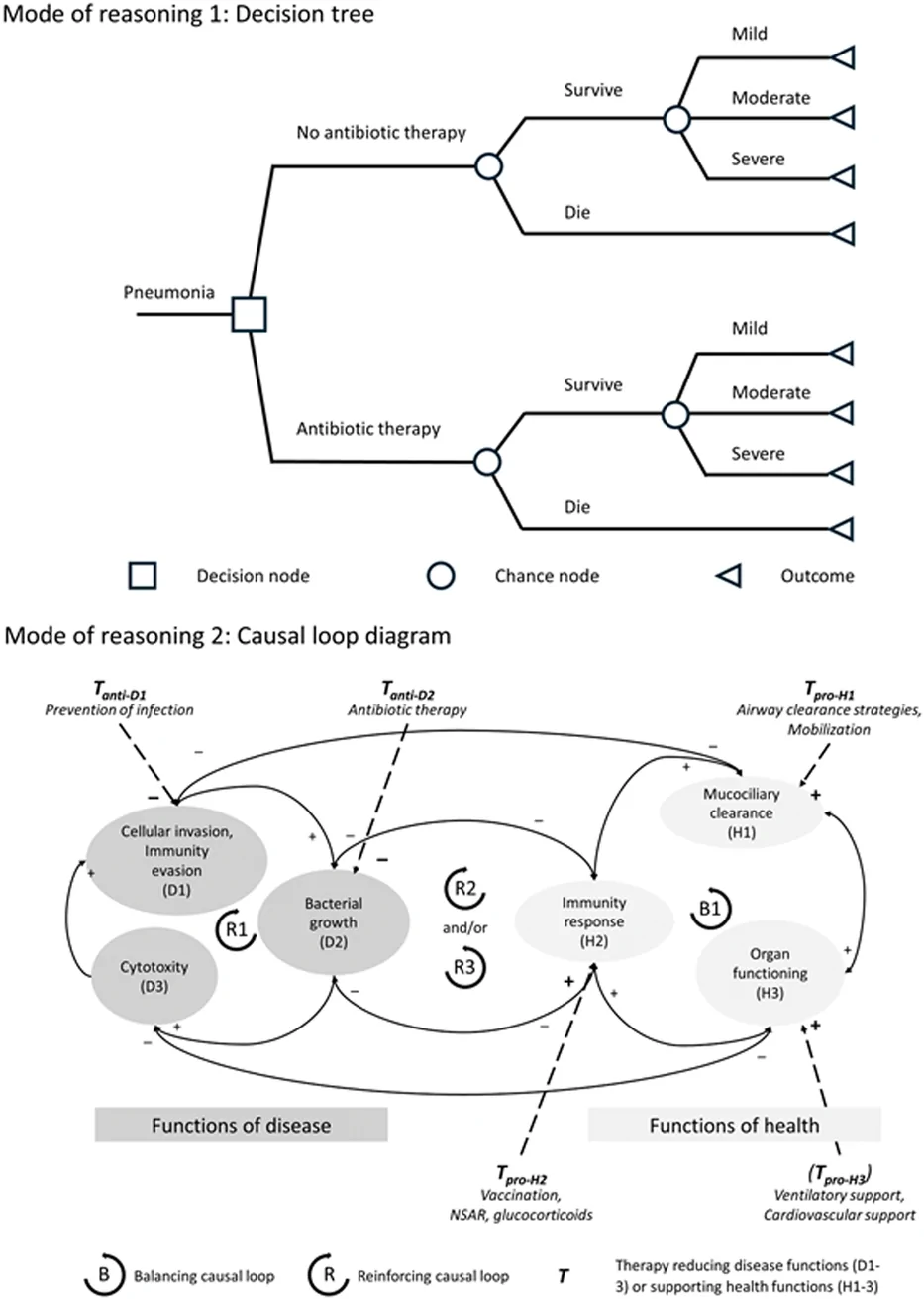
Two modes of therapeutic reasoning: decision trees and causal loop diagram. The mode of reasoning 1 (decision tree) conceptualizes therapeutic reasoning as a series of static choices among predefined pathways and intervention options. This mode is grounded in retrospective evidence and positions the clinician in the role of a “Bayesian statistician.” In this mode, decision‐making is viewed as a ‘back‐stage’ planning process that remains temporally and operationally distinct from the therapeutic action itself, typically unfolding across structured intervals such as individual consultations. In contrast, the mode of reasoning 2 (causal loop diagram) frames therapeutic reasoning as a dynamic, evolving process driven by causal feedback loops between the interconnected domains of disease, resilience, and health. Drawing on complex adaptive systems theory, this mode casts the clinician as a “reflective practitioner.” This mode emphasizes real‐time adaptation and reflection‐in‐action, viewing therapy as a continuous iterative response to emerging system behaviors rather than a mere selection of fixed, linear options.

The second tradition draws upon cognitive psychology and systems thinking. Schön's reflective practitioner emphasizes reflection‐in‐action and reflection‐on‐action as continuous dialog with unfolding situations [26]. Research on clinical reasoning highlights adaptive expertise, contextual sensitivity, and pattern recognition under uncertainty [27, 28, 29, 30, 31]. Systems‐oriented representations, including causal‐loop diagrams, emphasize feedback dynamics and emergent change (Figure 1, lower panel) [32].

These approaches reflect two distinct ontologies of clinical action: optimization among predefined options, as characterized by reasoning along decision trees, versus navigation within evolving systems, as envisioned by reasoning within a complex network of causally interconnected functions of health and disease. If therapy is conceptualized primarily as selection, reasoning focuses on choosing the correct option at a given decision point. Conversely, if therapy is understood as action within dynamic systems, reasoning centers on anticipating trajectories, recognizing emerging instability, and continuously reprioritizing goals in response to system change instead. This latter perspective requires a theoretical framework that accounts for adaptation, self‐regulation, and feedback within evolving systems. Complex adaptive systems theory provides such a framework.

## 4 Complex Adaptive Systems Theory as a Paradigm

Complex adaptive systems theory emerged across multiple disciplines during the second half of the twentieth century to explain how interacting components generate adaptive, self‐regulating, and emergent system behavior. Complex adaptive systems are characterized by continuous adaptation to changing conditions, driven fundamentally by feedback loops: negative feedback can stabilize system states, whereas positive feedback can amplify perturbations and drive transitions to new states. Complex adaptive systems theory has been widely applied across biological, ecological, social, and organizational domains to understand adaptation, self‐organization, resilience, and transitions between system states [33, 34].

In medicine, complex adaptive systems theory has been applied to health and disease, clinical practice, healthcare organizations, and health systems, where biological, individual, social, and organizational processes interact dynamically [34, 35]. Biological organisms can be conceptualized as complex adaptive systems in which physiological processes continuously adapt via feedback mechanisms, while healthcare systems exhibit emergent behavior driven by interactions among patients, professionals, and organizations [36, 37, 38, 39]. Complex adaptive systems theory has also informed medical education, including learning environments, clinical practice, interprofessional collaboration, and curriculum development [33]. Similarly, therapeutic processes have been conceptualized through the lenses of complex adaptive systems theory to capture the intricate dynamics between disease processes, physiological responses, and therapeutic interventions [35]. However, to our knowledge, complex adaptive systems theory has not yet been explicitly developed as a conceptual foundation for understanding and teaching therapeutic reasoning as the longitudinal regulation of evolving clinical trajectories. Our framework addresses this gap by extending this established perspective to a distinct dimension of clinical reasoning: how clinicians anticipate, monitor, and regulate changing clinical trajectories through targeted therapeutic action.

## 5 Disease Through the Lens of Complex Adaptive Systems Theory

Applying this perspective to disease requires a shift from viewing dysfunction as isolated and static toward conceptualizing it as evolving trajectories shaped by adaptation, resilience, and feedback. The human organism comprises interdependent functions and subsystems whose interactions continuously shape system behavior over time. For clinical practice, this perspective shifts attention from deliberative analysis of disease states toward situated action within evolving patient encounters, ranging from clinical consultations to complex surgical procedures. For medical education, this implies that therapeutic competence must encompass the capacity to interpret system behavior over time, anticipate emerging patterns, and modulate interventions iteratively rather than relying on the static matching of treatments to diagnoses.

Responses of the human organism to disease typically manifest as resistance, adaptation, or transformation. Early type 2 diabetes, for example, illustrates compensatory resistance: peripheral insulin resistance is initially offset by enhanced β‐cell insulin secretion and limited hypertrophy, maintaining near‐normoglycemia [40]. Chronic heart failure exemplifies adaptation, where sustained hemodynamic stress triggers neurohormonal activation and myocardial remodeling to preserve cardiac output [41]. Finally, post‐stroke recovery reflects transformation, involving activity‐dependent cortical reorganization that reshapes functional networks beyond the original circuitry [42].

Disease trajectories typically display non‐linear characteristics. For instance, sepsis often presents with deceptive stability before rapidly deteriorating via self‐amplifying feedback loops [43]. Similarly, bronchial asthma alternates between stability and acute bronchoconstriction [44]. Such critical transitions occur when small perturbations trigger cascading failures across biological system functions [37, 39]. Acute kidney injury, particularly when progressing to multiorgan failure, exemplifies such tipping point dynamics [45]. Early warning signals – such as increased variance in vital signs [46], reduced heart rate variability [47], delayed creatinine recovery [45], or escalating symptom autocorrelation in psychiatric relapse [48, 49] – serve as indicators of reduced resilience and critical slowing down.

## 6 The Teaching of Therapeutic Reasoning as Navigation of Trajectories

As disease unfolds within a dynamic human organism, therapy can be conceptualized not merely as the application of treatment, but as an intervention within an evolving system. Medical treatment may be conceived as the deliberate navigation of two coupled complex adaptive systems: the human organism and the underlying pathological process. It aims to selectively influence specific functions within this multi‐layered feedback‐regulated system and to modulate their magnitude in a therapeutically favorable direction over time.

From the therapeutic perspective, three groups of function are particularly salient: pathogenic functions that drive disease progression, resilience functions that counteract physiological disturbances, and health functions that sustain systemic integrity. Consequently, the objective of therapy extends beyond the attenuation of pathology and also includes the reinforcement of resilience and the restoration of homeostatic health functions.

Analogous to Lotka‐Volterra predator‐prey dynamics [50], disease processes may propagate at the expense of physiological health functions, while resilience mechanisms constrain and recalibrate this expansion. This analogy elucidates a principle often left implicit in clinical care: therapeutic action does not merely eliminate a static entity, but rather perturbs a shifting equilibrium (Figure 1, lower panel).

Within this framework, therapeutic reasoning is defined as the navigation of causally interconnected functions that shape trajectories over time. Upward trajectories represent recovery, while downward trajectories indicate clinical deterioration. Importantly, early improvement may reflect therapy‐dominant modulation, whereas sustained recovery often depends on endogenous resilience reclaiming regulatory control. Destabilizing events – including adverse responses to therapy – can abruptly reverse a trajectory direction, shifting the system into a new clinical phase (Figure 2). For learners, these inflection points often represent moments of greatest uncertainty and, therefore, provide the richest opportunities for developing adaptive expertise [51].

**Figure 2:**
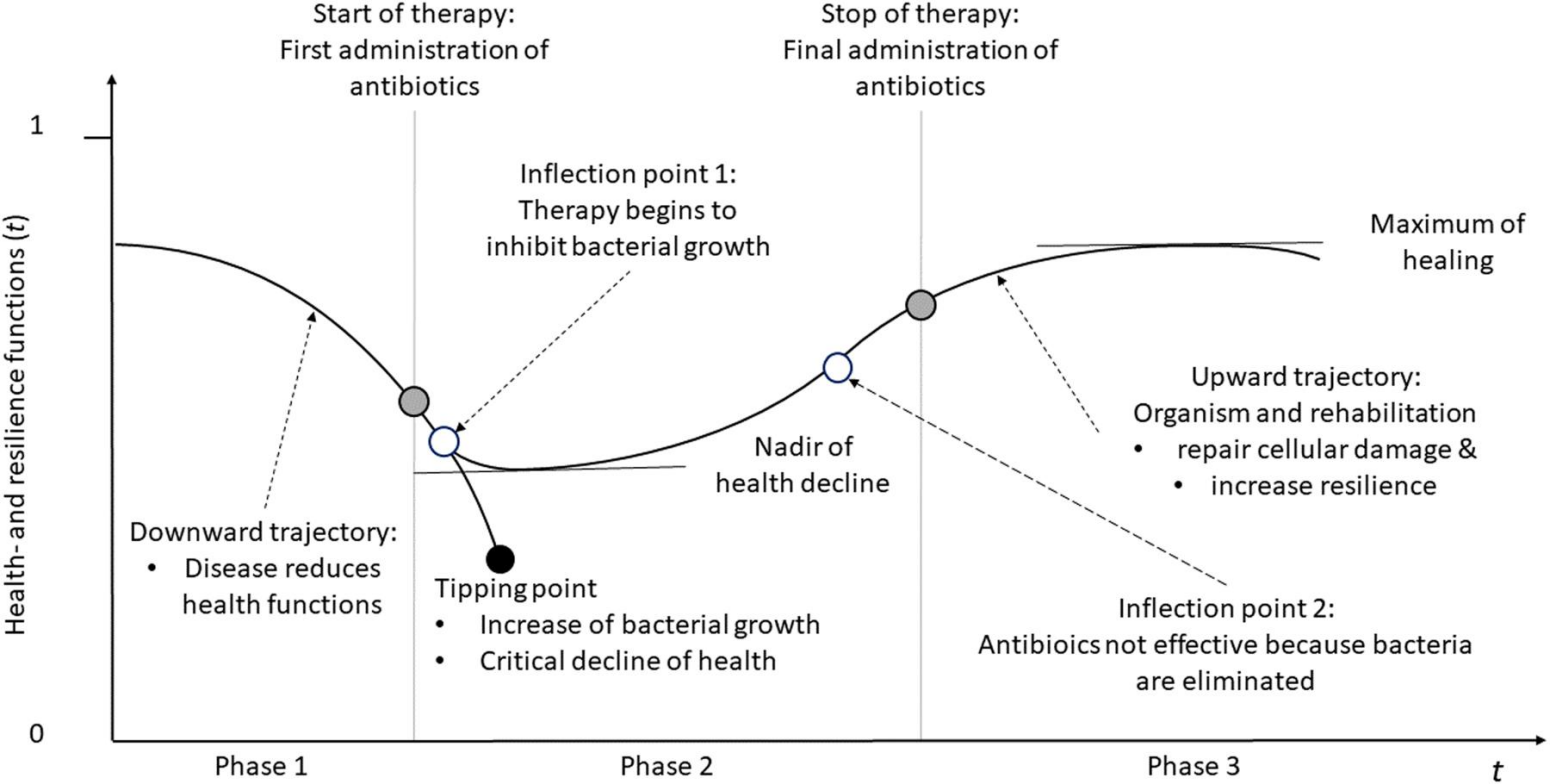
Navigating clinical trajectories. Medical treatment can be understood as navigating a patient's clinical trajectory which emerges from the dynamic interaction between the human organism and pathological processes as coupled with complex adaptive systems. Effective therapeutic reasoning requires anticipating how interventions influence systemic homeostasis, recognizing critical tipping points, and adapting priorities as trajectories evolve. This framework is organized into three dynamic phases. Phase 1: Disease‐dominant decline – progressive deterioration that necessitates early recognition of downward trends. Phase 2: Therapy‐driven modulation – interventions begin to attenuate pathology and produce gradual clinical improvement, while also carrying the risk of further decline due to unintended effects of treatment. Phase 3: Resilience‐dominant recovery – adaptive regeneration leads to stabilization and supports relapse prevention.

Therapeutic reasoning, therefore, necessitates the anticipation of how specific interventions modulate systemic homeostasis, the identification of critical thresholds where compensatory mechanisms fail, and the early recognition of clinical trajectories that mandate a reprioritization of care. To address these complexities, we propose a didactic structure organized into three dynamic phases (Figure 2): phase 1) Disease‐dominant deterioration (where pathophysiological processes outpace physiological reserves), phase 2) Therapy‐mediated modulation (active stabilization and redirection of systemic variables through therapeutic measures), and phase 3) Resilience‐driven recovery (the transition towards endogenous restoration with the re‐establishment of physiological equilibrium).

The management of severe pneumonia exemplifies these dynamics, specifically the critical inflection points between clinical phases and the necessity of reprioritizing care during acute destabilizing events such as anaphylaxis (Figure 2). Since this navigation of clinical trajectories occurs across medical specialties from internal medicine to surgery and intensive care, this framework serves as a cross‐cutting conceptual anchor within otherwise fragmented curricula with respect to medical specialties.

## 7 Suggestions for Teaching Strategies of Therapeutic Reasoning

Teaching therapeutic reasoning necessitates more than merely adding a new tool. It requires rendering the temporal and regulatory dimensions of therapy cognitively implicit and pedagogically scaffolded within practice‐oriented learning environments [27, 28]. Rather than imposing an additional layer of theory, the following strategies delineate core objectives for different learners and educational contexts conductive to cultivating therapeutic reasoning. To this end, we propose three complementary instructional strategies to operationalize the core dimensions of systems responsiveness and therapeutic agility throughout the continuum of medical training.

Firstly, causal loop diagraming is particularly suited for undergraduate and early postgraduate learners within classroom or small‐group settings [52]. It enables students to externalize the feedback structures that link disease processes, resilience mechanisms, and health functions. By identifying self‐reinforcing vicious cycles and mapping potential intervention points, learners are enabled to translate physiological and pathophysiological knowledge into a coherent therapeutic logic. Furthermore, recognizing endogenous resilience mechanisms encourages a shift in perspective from mere disease suppression toward the amplification of resilience. Such exercises reorganize compartmentalized biomedical knowledge into dynamic relational models, thereby explicitly bridging the gap between preclinical basic sciences and clinical clerkships (Figure 1, lower panel). Workplace‐based assessments like Clinical Work Sampling [53], the Mini‐Clinical Evaluation Exercise (Mini‐CEX) and the Direct Observation of Procedural Skills (DOPS) can effectively capture the early externalization of these models during supervised clinical tasks [54].

Secondly, the construction of trajectories operationalizes temporal awareness and is particularly applicable in clinical clerkships, ward‐based teaching, and simulation‐based training. By delineating prototypical disease trajectories—spanning deterioration, stabilization, and recovery—learners refine their capacity to time therapeutic interventions, including initiation, combination, tapering, or discontinuation, in accordance with the current systemic state. The visualization of therapy‐response curves facilitates a more sophisticated anticipation of response variability [55]. Consequently, structured timeline mapping reinforces the temporal dimension in therapeutic reasoning [55]. Simulations that incorporate typical tipping points or nonlinear shifts provide advanced undergraduate students and residents with opportunities to rehearse adaptive adjustments under conditions of evolving uncertainty [28]. In this manner, trajectory‐based learning fosters therapeutic agility, that is the ability not only to select the correct intervention but also to recalibrate therapy in response to unfolding clinical trajectories. Given that trajectory‐based reasoning is visually intuitive and easily communicable, it is well‐suited for integration into ward rounds [56], interdisciplinary case conferences, and briefings or debriefings following complex surgical interventions [57, 58]. Finally, the use of Mini‐CEX, DOPS, and extended Script Concordance Tests could render these trajectory‐based therapeutic discussions observable and assessable [59].

Thirdly, explicit cultivation of systems responsiveness is most effectively realized within advanced clinical training and interprofessional contexts. In these settings, the learning focus shifts from the construction of theoretical models to the real‐time detection of clinical inflection points, tipping dynamics, nadirs of decline, and peaks of recovery in actual patients. Teaching therapeutic intervention as an ongoing dialog with the interconnected systems of disease, human health, and therapeutic actions encourages learners to interpret subtle indicators – such as critical slowing down, increased variance, or flickering – that may signal shifts in systemic stability. To render these complex interpretive processes explicit, discussable and assessable, several structured educational strategies can be employed. Reflective debriefings, morbidity and mortality conferences, and think‐aloud protocols [60] serve to externalize therapeutic reasoning. When combined with workplace‐based assessments such as Mini‐CEX and DOPS [54], portfolios documenting trajectory management over repeated patient encounters not only provide a robust assessment framework but also actively support learners’ longitudinal development and reflection [61].

## 8 Reframing the Education of Therapeutic Reasoning

Therapeutic reasoning is most effectively taught when fully integrated into existing curricular architectures, rather than treated as a separate, discrete capability. Seminar‐ and skills‐lab exercises—such as guideline critique and role‐play of shared decision‐making conversations—can transform guidelines from prescriptive rules into dialogical resources that invite critical engagement [62, 63]. Complementing these methods, systems‐oriented tools foster flexible, context‐sensitive judgment across specialties, particularly within longitudinal integrated clerkships and postgraduate training [62, 63].

By conceptualizing therapeutic reasoning as navigation along clinical trajectories, medical education extends beyond the selection of treatments to nurture temporal awareness, responsiveness to feedback, and adaptive reprioritization [64, 65, 66].

## 9 Curricular Integration Across Training Stages

Conceptualizing therapeutic reasoning as the navigation of clinical trajectories necessitates a fundamental reconfiguration of how therapy is theoretically conceptualized and didactically sequenced across the continuum of medical training. This shift implies that therapeutic interventions should not be taught as static, isolated events but rather as dynamic processes requiring ongoing adjustment within a longitudinal patient narrative. Consequently, curricula must be structured to support the progressive development of these navigational skills ensuing that the employed didactic strategies align with the increasing complexity of clinical decision‐making from undergraduate education through to professional mastery.

Undergraduate medical curricula remain primarily structured around diagnostic classification. Consequently, therapeutic content is often presented as algorithmic application of therapeutic guidelines, which may inadvertently reinforce a static decision‐making paradigm. This contributes to the documented challenges learners face in treatment‐focused reasoning compared to diagnostic reasoning [7, 67]. A trajectory‐based framework for therapeutic reasoning provides an early cognitive scaffold that precedes the acquisition of detailed specialty‐specific knowledge. By introducing foundational trajectory concepts – such as directionality, feedback dynamics, tipping points, delayed effects, and phase transitions – prior to extensive instruction in pharmacology or specialty‐specific interventions, curricula can foster temporal literacy. This is defined as the capacity to conceptualize therapy as a process of longitudinal regulation rather than a single, discrete decision. Visual tools such as causal loop diagrams allow novices to engage with complex regulatory logic without requiring deep specialty expertise. Introducing trajectory‐based language early in medical education may normalize longitudinal thinking before students enter specialty training [68].

In postgraduate medical education, particularly in internal medicine, emergency medicine, intensive care, and general practice, trainees encounter authentic longitudinal care contexts where clinical trajectories evolve over time. However, adaptive reprioritization is frequently modeled implicitly rather than explicitly. Consequently, deviation from an initial therapeutic plan may be misinterpreted as clinical uncertainty rather than as a hallmark of expert reasoning [69, 70]. To recalibrate this professional norm, trajectory awareness should be systematically integrated into clinical discourse, including case presentations, ward rounds, and morbidity and mortality conferences. Supervisors can facilitate this by explicitly framing discussions around anticipated clinical courses, i.e., trajectory direction, signals of instability, and the timing of phase transitions. Probing questions—such as “What trajectory did we expect?”, “Which signals indicated a shift?”, and “When should goals have been recalibrated?”—serve to reposition therapeutic agility. Through this approach, postgraduate education moves from evaluating isolated correctness of therapeutic decisions toward evaluating regulatory adaptation of treatment over time.

At the level of continuing professional development, trajectory navigation corresponds directly to the realities of multimorbidity, polypharmacy, and the longitudinal management of chronic disease. Managing interacting clinical conditions requires balancing competing regulatory demands across organ systems and temporal horizons, a challenge that remains inadequately addressed by disease‐specific clinical training formats [71]. In these situations, therapeutic agility becomes a central element; clinicians must continuously recalibrate treatment intensity, redefine therapeutic goals, and transition from disease‐centric attenuation toward the support of physiological and psychological resilience as a patient's systemic states evolve. Across the continuum of medical education, the trajectory paradigm thus serves not as a supplementary curricular element, but as a longitudinal organizing principle. This framework aligns foundational sciences, clinical application, and lifelong learning within a unified logic of dynamic regulation.

## 10 Faculty Development for Therapeutic Reasoning

To integrate the approach of trajectory navigation into therapeutic reasoning within medical curricula, faculty must first conceptualize therapy as the longitudinal regulation of a biological system. Hence, faculty development should follow a structured progression from conceptual understanding of trajectory navigation to scaffolding techniques to support learners across supervisors’ career stages. Supervisors need to internalize the fundamental epistemic shift from viewing therapeutic action as the navigation of clinical trajectories rather than the mere execution of therapeutic guidelines. Faculty development workshops using clinical vignettes can introduce feedback loops, phase transitions, and recalibration triggers [72].

Effective instruction how to teach therapeutic reasoning according to the new framework of clinical trajectory navigation is enabled through three distinct career‐stage‐appropriate techniques: (1) Early career‐faculty (novice supervisors) need to focus on verbalization of trajectory anticipation during bedside teaching. By explicitly staging expected outcomes (e.g., “We anticipate clinical stabilization within 48 h; persistent fever would signal a reversal”), Supervisors employ cognitive apprenticeship to make tacit regulatory logic observable to learners [73]. (2) Mid‐career faculty (experienced clinicians) will have to transition their supervision to trajectory‐based feedback during workplace‐based assessments such as Mini‐CEX or DOPS [54]. Supervisors should use targeted questioning (e.g., “What clinical trajectory did you anticipate? “ or “At what point would you reprioritize your intervention?”), complemented by role‐playing to rehearse these reasoning patterns. (3) Advanced faculty (senior clinicians) should model systems responsiveness during complex case reviews and morbidity and mortality conferences. Learners' development can be supported by longitudinal 360° feedback and comprehensive teaching portfolio reviews. These strategies can be integrated seamlessly into existing education structures including formal teaching observation, grand rounds, and peer coaching. The staged developmental progression ensures that supervisors can effectively scaffold therapeutic reasoning for trainees in different educational stages, ultimately transforming this conceptual paradigm into sustainable clinical practice.

## 11 Limitations and Potential Risks

The proposal to integrate clinical trajectory navigation into therapeutic reasoning education faces relevant challenges that transcend mere didactic adjustment. A fundamental concern regards epistemic legitimacy. Although complex adaptive systems theory offers a sophisticated framework for interpreting biological and clinical dynamics, its practical application to bedside decision‐making lacks a comprehensive theoretical foundation. The assertion that therapeutic expertise is defined by the navigation of non‐linear trajectories is conceptually appealing, but susceptible to overgeneralization. Not all clinical processes exhibit meaningful non‐linearity at the scale relevant to decision‐making in daily practice [74]. Rather, much of therapeutic decision‐making remains protocol‐driven and follows statistically stable, textbook‐like predictable courses [75]. Consequently, overemphasizing systemic volatility may skew learners' perceptions of routine care and inflate the apparent frequency of critical tipping points.

Another limitation is that the framework currently lacks rigorous empirical validation regarding measurable educational outcomes. While systems thinking and adaptive expertise are well documented within educational theory, direct evidence demonstrating that explicit trajectory‐based training improves diagnostic precision, reduces adverse events, or optimizes patient outcomes is lacking. In the absence of such evidence, the proposal remains theoretically driven rather than empirically substantiated. Within the context of evidence‐based medical education, this lack of outcome‐oriented validation constitutes a significant vulnerability [76, 77].

Trajectory‐based therapeutic reasoning may inadvertently exacerbate unwarranted variation in clinical care. Should learners internalize the concept of adaptive reprioritization without robust grounding in evidence‐based standards, there is a risk that variability will expand rather than become more precisely calibrated. Distinguishing between context‐sensitive adjustment and idiosyncratic deviation remains difficult to operationalize and even more challenging to assess. Within highly regulated healthcare environments, such ambiguity is likely to encounter institutional resistance.

Furthermore, the clinical environments, which are characterized by time pressure, fragmented information, and competing demands, could constitute a limitation for teaching clinical trajectory navigation. Consequently, the expectation that supervisors can consistently articulate anticipated trajectories and regulatory inflection points may be unrealistic. Additionally, faculty modeling is itself subject to bounded rationality; many adaptive decisions are intuitive and post hoc rationalizations could oversimplify the actual underlying cognitive processes. Thus, the assumption that therapeutic reasoning can always be externalized into a coherent trajectory narrative may underestimate the tacit and heuristic nature of expert performance.

Moreover, the framework presupposes a level of systemic coherence in patient care that may not exist. Many healthcare systems are fragmented, with discontinuities between inpatient, outpatient, and community settings. Instructing learners to apply longitudinal thinking in environments that structurally undermine longitudinal care may result in cognitive dissonance rather than improved therapeutic reasoning.

These limitations suggest that trajectory‐based therapeutic reasoning education should be framed as a theoretical enrichment rather than a paradigm replacement. Trajectory‐based therapeutic reasoning requires rigorous empirical testing, contextual adaptation, and institutional alignment. The model's theoretical plausibility does not guarantee its universality, scalability, or demonstrable impact. A responsible next step for using this framework to teach therapeutic reasoning therefore lies not in premature claims for broad implementation but in focused pilot studies and critical evaluation.

## 12 Reframing Therapeutic Reasoning in Medical Education

Therapeutic reasoning occupies a central yet conceptually demanding position in medical education. Frequently, therapeutic reasoning is reduced to the selection of appropriate interventions in accordance with clinical guidelines or evidence hierarchies. We propose a reframing of this concept, arguing that therapeutic reasoning in complex clinical environments should be understood not merely as a process of decision selection, but as a form of longitudinal system regulation.

This shift in perspective moves the analytical focus from discrete decision points toward evolving trajectories. Traditional didactic models tend to emphasize the correctness a particular pharmacological agent, procedure, or management plan at a specific moment in time. While indispensable, such a focus abstracts therapeutic action from the temporal dynamics in which it naturally unfolds. Within complex adaptive biological and psychosocial systems, interventions do not simply solve problems; instead, they perturb systems whose responses may be delayed, amplified, dampened, or redirected through feedback loops. Under these conditions, therapeutic reasoning involves the anticipation of trajectories, the continuous monitoring of system signals, and the recalibrating of interventions as system states evolve.

Conceptualizing therapeutic reasoning through the lens of non‐linear trajectories does not displace the principles of evidence‐based medicine. Guidelines remain essential epistemic anchors. However, they primarily address which actions to take under specified conditions and are often less explicit regarding how those system conditions change over time. A trajectory‐oriented framing reveals that escalation, de‐escalation, sequencing, and timing are intrinsic components of therapeutic reasoning rather than secondary adjustments. Ultimately, this approach highlights that therapeutic reasoning continues well beyond the initial therapeutic decision, as clinicians must interpret system responses and reconfigure interventions accordingly.

Importantly, this reframing is not universally applicable across all domains of care. In stable and predictable contexts, therapeutic reasoning may indeed approximate static application. Yet in settings characterized by multimorbidity, interacting pathophysiology, and clinical instability, the limits of purely static decision models become apparent. In such instances, reasoning is inseparable from temporal navigation.

The contribution of a trajectory‐based perspective on therapeutic reasoning therefore lies in conceptual clarification. It names and structures a dimension of therapeutic reasoning that is already present in expert practice but insufficiently articulated in curricula. By integrating insights from complex adaptive systems theory into medical education discourse, the framework does not introduce a new clinical act; rather, it refines how an existing one is understood.

## Author Contributions

Yskert von Kodolitsch designed and wrote the manuscript. Tilman Dreyer, Thomas Wick, and Sigrid Harendza discussed the design, and critically reviewed and edited the manuscript.

## Funding

The authors have nothing to report.

## Ethics Statement

The authors have nothing to report.

## Conflicts of Interest

The authors declare no conflicts of interest.

## Data Availability

Data sharing is not applicable to this article as no datasets were generated or analyzed during the current study.
